# Time-of-Day Dictates Transcriptional Inflammatory Responses to Cytotoxic Chemotherapy

**DOI:** 10.1038/srep41220

**Published:** 2017-01-24

**Authors:** Jeremy C. Borniger, William H. Walker II, Monica M. Gaudier-Diaz, Curtis J. Stegman, Ning Zhang, Jennifer L. Hollyfield, Randy J. Nelson, A. Courtney DeVries

**Affiliations:** 1 Department of Neuroscience, Neuroscience Research Institute, and Behavioral Neuroendocrinology Group, The Ohio State University, Wexner Medical Center, Columbus, OH 43210 USA

## Abstract

Many cytotoxic chemotherapeutics elicit a proinflammatory response which is often associated with chemotherapy-induced behavioral alterations. The immune system is under circadian influence; time-of-day may alter inflammatory responses to chemotherapeutics. We tested this hypothesis by administering cyclophosphamide and doxorubicin (Cyclo/Dox), a common treatment for breast cancer, to female BALB/c mice near the beginning of the light or dark phase. Mice were injected intravenously with Cyclo/Dox or the vehicle two hours after lights on (*zeitgeber* time (ZT2), or two hours after lights off (ZT14). Tissue was collected 1, 3, 9, and 24 hours later. Mice injected with Cyclo/Dox at ZT2 lost more body mass than mice injected at ZT14. Cyclo/Dox injected at ZT2 increased the expression of several pro-inflammatory genes within the spleen; this was not evident among mice treated at ZT14. Transcription of enzymes within the liver responsible for converting Cyclo/Dox into their toxic metabolites increased among mice injected at ZT2; furthermore, transcription of these enzymes correlated with splenic pro-inflammatory gene expression when treatment occurred at ZT2 but not ZT14. The pattern was reversed in the brain; pro-inflammatory gene expression increased among mice injected at ZT14. These data suggest that inflammatory responses to chemotherapy depend on time-of-day and are tissue specific.

The toxicity of over 40 anticancer drugs, including cytostatics, cytokines, and targeted biological agents, is largely modified by circadian timing^1^. The DNA intercalator doxorubicin^2^ and the alkylator cyclophosphamide^3^ show marked circadian variation in toxicity and tolerability. In the case of doxorubicin, these circadian effects persist in constant darkness or constant light, eliminating the covariate of masking by darkness or light^4^. As circadian physiology between nocturnal rodents and diurnal humans is nearly 12 h out of phase, drug chronopharmacology typically displays opposite patterns between these species^5^.

Cancer chronotherapy depends on administration of treatment at times that coincide with optimal drug metabolism and effects on cell cycle progression, DNA repair, and apoptosis^1^. Additionally, several studies have demonstrated that peripherally administered cancer chemotherapeutics cause a peripheral and central inflammatory response^6^,^7^,^8^,^9^,^10^. Inflammatory cytokines (e.g., IL-1β, IL-6, and TNF-α) display a circadian rhythm in production and secretion, peaking during the early inactive phase coinciding with sleep onset, although organ- and cell-type deviations exist^11^,^12^,^13^,^14^. Scheving and colleagues demonstrated a several-fold greater efficacy of doxorubicin-cyclophosphamide in male mice treated at ZT13 [*zeitgeber* time (ZT); 1 h into active phase] as compared to ZT1 (1 h into inactive phase); the mechanisms may relate to enhanced cytokines production at ZT1, that could then both disrupt the immune response of the host and accelerate cancer growth^15^.

It remains unknown whether inflammatory responses to chemotherapeutics are altered by the timing of drug administration throughout the day, and whether the inflammatory response is linked to ‘time-of-day dependent’ side effects of treatment. One recent study examined transcript changes in the liver of mice administered various doses of cyclophosphamide (Cyclo) during the mid-day and mid-night (ZT 8 and ZT 20)^16^. Genes involved in immune responses were significantly upregulated by cyclophosphamide only in the group that was administered the drug during the light phase, suggesting that hepatic immune responses to cyclophosphamide are under circadian control. It is also unknown whether similar responses occur centrally, in other organs, or with different chemotherapeutic regimens. We hypothesized that time-of-day influences inflammatory responses to cytotoxic chemotherapy. To test this hypothesis, we administered a cocktail of cyclophosphamide/doxorubicin (Cyclo/Dox; IV) at two different times of day (shortly after onset of light, and shortly after onset of dark) to female ovariectomized mice, and collected tissue/serum 1, 3, 9, and 24 hours later (experimental design: Fig. 1). We predicted that mice administered doxorubicin/cyclophosphamide chemotherapy at the beginning of their normally inactive phase (ZT 2) would display an exaggerated inflammatory response compared to mice injected at the beginning of their normally active phase (ZT 14).

## Methods

### Animals

Adult female BALB/c mice (>8 wks; Charles River Laboratories, Wilmington, MA, USA) were used in the experiments described herein. All mice were allowed to acclimate to our facility for 1 wk after arrival, and then were ovariectomized to eliminate the covariate of acute ovarian failure in response to chemotherapy^17^,^18^. All surgeries were conducted using aseptic technique by an experienced surgeon (NZ), and mice were supplied analgesia (0.05 mg/kg buprenorphine, SC) and post-operative warmth (cage placement on a heating pad) until mobile. Ibuprofen (~30 mg/kg) was supplied in the drinking water for 5 days following surgery for further analgesia. Mice were group housed (4/cage) for 2 wks prior to experimental assignment. Mice were supplied *ad libitum* food (Harlan Teklad #7912) and filtered tap water, as well as a cotton nestlet. Cages were changed weekly. Mice were maintained on a 12:12 light/dark cycle. Body mass measures were obtained on the day prior to injection, and then at tissue collection 1, 3, 9, and 24 h post-injection. All experiments were approved and carried out in accordance with guidelines set by The Ohio State University Institutional Animal Care and Use Committee (IACUC).

### Agent Administration

At ZT 2 (2 h after lights ON) or ZT 14 (2 h after lights OFF), mice were brought into an adjacent procedure room, briefly restrained in a conical tube, and injected intravenously (tail vein) with a cocktail of 13.5 mg/kg doxorubicin and 135 mg/kg cyclophosphamide or saline vehicle as previously described^10^. This drug cocktail is commonly used as adjuvant treatment for localized breast cancer, as well for the treatment of metastatic disease^19^. Dosing was determined from 75% of the human equivalent dose based on a body surface area equation^20^. Injections at ZT 14 were completed under dim (<5 lux) red light to prevent circadian disruption by short-wavelength light.

### Tissue Collection

Mice were rapidly euthanized via cervical dislocation and decapitation 1, 3, 9, and 24 h after agent administration. Whole brain, spleen and liver were collected on ice into 1.5 mL polypropylene tubes containing RNA-Later Reagent (Qiagen), and subsequently stored at −80 °C until RNA extraction or other downstream analyses. Whole blood was collected, centrifuged at 4000 × g for 25 min, and serum supernatant was subsequently stored at −80 °C until assayed.

### Cytokine Multiplex Assay

To examine cytokine and chemokine concentrations in serum, a multiplex inflammatory panel was conducted (V-Plex proinflammatory panel 1, Meso-Scale Discovery, Rockville, Maryland, USA) according to the manufacturer’s instructions. This kit measures protein levels of IFN-γ, IL-10, IL-12p70, IL-1β, IL-2, IL-4, IL-5, IL-6, KC/GRO (CXCL1), and TNF-α. A subset of the samples (111/128) had sufficient serum to be included in the multiplex assay; n ≥ 4/group/timepoint).

### Real-Time Quantitative Polymerase Chain Reaction (RT-qPCR)

RNA was extracted using TriZol reagent (Life Technologies, Thermo Fisher Scientific Inc.) according to the manufacturer’s instructions. RNA pellets were resuspended in sterile, RNase-free water and quality and quantity were determined using a spectrophotometer (NanoDrop, Thermo Fisher Scientific Inc.). cDNA was synthesized using M-MLV reverse transcription. 20 ng of subsequent cDNA template was used in each qPCR reaction. Inventoried primer/probe pairs from Applied Biosystems (Life Technologies) were used (information available in Table 1). Probes that span exons were chosen to prevent amplification of residual gDNA. Taqman Fast Advanced Master Mix (Life Technologies) containing AmpliTaq Fast DNA polymerase was used in a 20 μL duplex reaction containing target primer/probes and one for the endogenous control eukaryotic 18 s rRNA. The 2-step RT-qPCR cycling conditions were: a holding stage of 95 °C for 20 s, 40 cycles of 95 °C for 3 s, and then 60 °C for 30 s. Relative gene expression was calculated using a relative standard curve, normalized to 18 s rRNA signal.

### Corticosterone Enzyme Immunoassay (EIA)

Serum corticosterone was determined using an enzyme immunoassay kit (DetectX, K014-H5, Arbor Assays, Michigan, USA). In brief, samples were diluted with dissociation reagent and assay buffer 1:100, and assayed in duplicate. Plates were read on a plate reader at 450 nm, and concentrations were determined using a 4-parameter logistic curve plotting unknowns against standard corticosterone values. This assay was run after using a significant amount of serum for the multiplex cytokine analyses described above, and therefore not all samples contained sufficient serum to be assayed for corticosterone (77/128). Therefore, the results presented provide only a snapshot of corticosterone concentrations at 24 h post-injection. The average intra-assay coefficient of variation was 8.17% and the inter-assay %CV was 7%.

### Statistical Analyses

Factorial ANOVAs with treatment, injection time, and time after injection as fixed factors were used. To control for circadian variation in gene expression, data were analyzed (and displayed) as fold change from time-matched vehicle controls. When significant *F* values were detected, post-hoc univariate ANOVAs were completed to examine time-point specific differences between groups. Data that did not show equal variance were subjected to non-parametric tests (i.e., Mann-Whitney U). Percent change in body mass measures from baseline between each group was compared using a paired-samples *t*-test. Values for each variable that fell ≥2 standard deviations outside of the group mean (Z-score) were considered outliers and removed from analyses *a priori*. For the spleen, liver, hypothalamus, and hippocampus, 3, 7, 4, and 4 samples (respectively) failed to amplify during PCR, and their data were therefore not analyzed. Outliers were determined within each experimental group (that is, split by time of injection (ZT 2 vs. 14), injection type (chemo vs. veh), and tissue collection time point (1, 3, 9, and 24 h later) resulting in 16 groups that were independently tested for aberrant values in each measure. After this, each group had between 6–8 animals for the spleen, 5–8 animals for the liver, and 5–8 animals for the brain per gene analyzed; no more than 1 animal/timepoint/treatment was excluded by Z-score analysis. Final sample sizes are listed in the figure legends. Body mass for one mouse was not recorded prior to sample collection and is therefore not included in the analysis. All statistics were completed using SPSS Statistics version 23 (IBM, Armonk, NY) and GraphPad Prism 5.0 (GraphPad Software, San Diego, CA). Mean differences were considered statistically significant when p ≤ 0.05.

## Results

### Time of Injection Alters Body Mass Loss After Cyclo/Dox Administration

**Figure 2:**
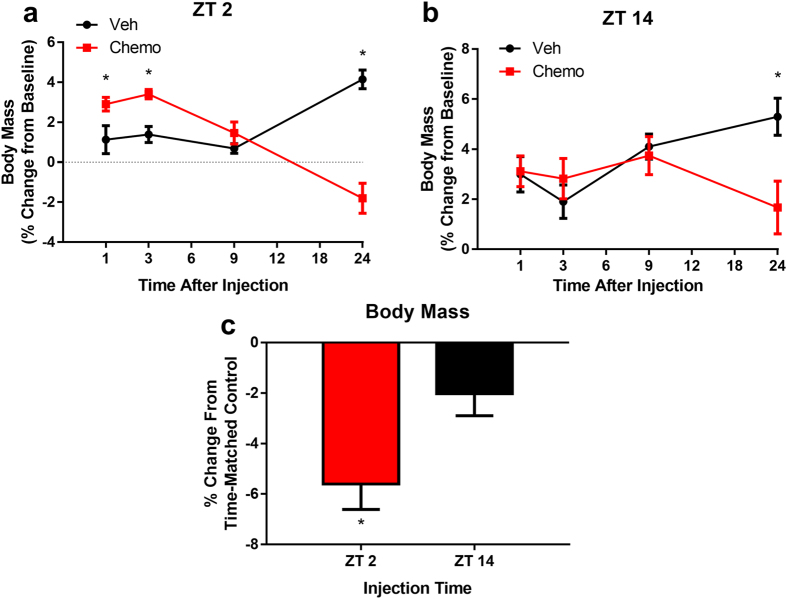
Time-of-day influences body mass loss following doxorubicin/cyclophosphamide administration. (**a**) Change in body mass after injection at ZT 2, and (**b**) ZT 14. (**c**) % change in body mass from time-matched vehicle treated mice at 24 h post-injection in animals injected at ZT 2 and ZT 14. ((N = 7–8/group/timepoint) Error bars represent SEM. *p < 0.05)).

Due to normal circadian variation in food intake and activity that can affect body mass, analyses were conducted on percent change in body mass from baseline. 24 h after agent administration, ZT 2 treated animals lost significantly more weight (−5.603 ± 2.85%) than their counterparts injected at ZT 14 (−2.02 ± 2.48%; *t* = 3.147, p = 0.016) compared to their time-matched vehicle controls (Fig. 2).

### Injection Time Alters Splenic Cytokine Transcription

**Figure 3:**
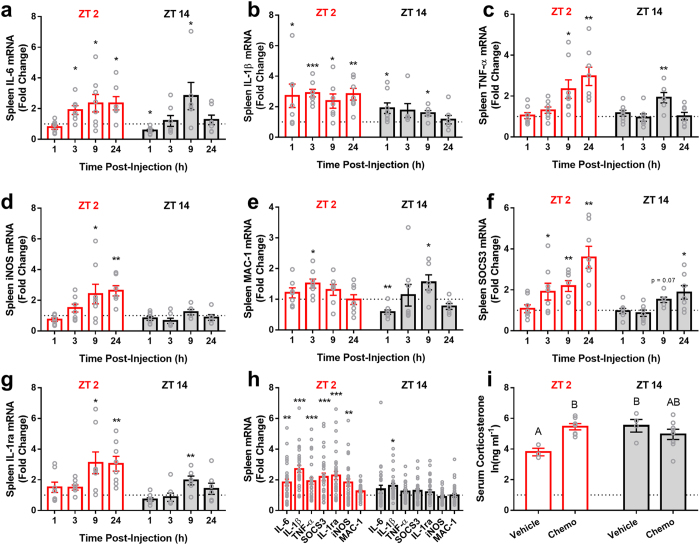
Time-of-day alters splenic cytokine expression in response to IV cyclophosphamide and doxorubicin. (**a**–**d**) Expression of the inflammatory cytokines IL-6, Il-1β, TNF-α, and iNOS were enhanced in mice receiving Cyclo/Dox at ZT 2 compared to those administered the same dose at ZT 14. (**e**) By contrast, expression of MAC-1 was unchanged. (**f**,**g**) Anti-inflammatory pathway transcripts (SOCS3 and IL-1ra) were simultaneously enhanced in mice administered Cyclo/Dox at ZT 2 compared to ZT 14. (**h**) Average gene expression values across the entire post-injection time frame, (**i**) serum corticosterone concentrations 24 following injection (N = 3–8/group; different letters denote statistical significance p < 0.05; Tukey HSD post-hoc test). (N = 6–8/group/timepoint for gene expression data; error bars represent S.E.M. *p < 0.05, **p < 0.01, ***p < 0.001 in relation to the time-matched vehicle control). The dotted line at y = 1 represents normalized vehicle values.

Cytokine transcription was measured in the spleen as an index of the peripheral immune response (Fig. 3). Chemo-treated animals increased gene expression of splenic IL-1β (*F*_*1,95*_ = 46.005, p < 0.0001), IL-6 (*F*_*1,97*_ = 11.97, p < 0.001), TNF-α (*F*_*1,101*_ = 22.551, p < 0.0001), SOCS3 (*F*_*1,101*_ = 32.86, p < 0.0001), IL-1ra (*F*_*1,100*_ = 22.164, p < 0.0001), iNOS (*F*_*1,98*_ = 6.243, p < 0.05), but not MAC-1 (aka ITGAM) (*F*_*1,98*_ = 1.461, p > 0.05). There was an interaction between injection time and treatment in IL-1β (*F*_*1,95*_ = 10.689, p < 0.005), TNF-α (*F*_*1,101*_ = 6.917, p < 0.01), SOCS3 (*F*_*1,101*_ = 11.6, p < 0.001), IL-1ra (*F*_*1,100*_ = 10.523, p < 0.005), iNOS (*F*_*1,98*_ = 10.539, p < 0.005), but not IL-6 (*F*_*1,97*_ = 1.308, p > 0.05) or MAC-1 (*F*_*1,98*_ = 1.293, p > 0.05) expression, such that animals injected at ZT 2 displayed elevated responses to chemotherapy injection compared to animals injected at ZT 14. Corticosterone concentrations were altered by injection time and treatment 24 h post-injection (*F*_*3,21*_ = 3.9, p < 0.05); corticosterone was elevated at ZT 14 compared to ZT 2 in vehicle-treated mice (Tukey HSD post-hoc; p < 0.05), and chemotherapy treatment increased circulating corticosterone concentrations in mice injected at ZT 2 (p < 0.05), but not ZT 14 (p > 0.05) (Fig. 3i). These data suggest that injection time has differential effects on the glucocorticoid response to chemotherapy treatment.

### Time of Injection Alters Central Cytokine Transcription

**Figure 4:**
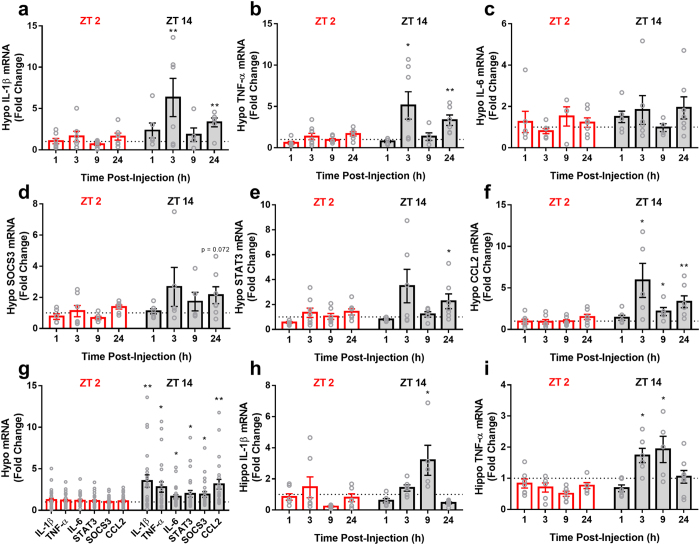
Time-of-day alters brain cytokine/chemokine expression in response to cytotoxic chemotherapy. Animals injected at ZT 14 displayed elevated hypothalamic (**a**) IL-1β, (B) TNF-α, (**c**) IL-6, (**d**) STAT3, (**e**) SOCS3, and (**f**) CCL2 expression over time in response to chemotherapy compared their counterparts injected at ZT 2 (**g**) Average hypothalamic values across the entire post-injection timeframe. (**h**) Hippocampal IL-1β and (**i**) TNF-α expression. (n = 5–8/group/timepoint; *p < 0.05, **p < 0.01 in relation to the time-matched vehicle control; Error bars represent S.E.M). The dotted line at y = 1 represents normalized vehicle values.

Central cytokine transcription was examined in the hypothalamus and hippocampus (Fig. 4). Chemotherapy increased hypothalamic IL-1β (*F*_*1,91*_ = 16.926, p < 0.0001), TNF-α (*F*_*1,92*_ = 11.591, p < 0.001), SOCS3 (*F*_*1,91*_ = 6.554, p < 0.05), STAT3 (*F*_*1,95*_ = 7.245, p < 0.01), IL-6 (*F*_*1,85*_ = 4.810, p < 0.05), and CCL2 (*F*_*1,93*_ = 22.336, p < 0.0001) expression. There also was an interaction between injection time and treatment in IL-1β (*F*_*1,91*_ = 11.469, p < 0.002), TNF-α (*F*_*1,92*_ = 8.16, p < 0.006), CCL2 (*F*_*1,93*_ = 18.846, p < 0.0001), SOCS3 (*F*_*1,91*_ = 7.087, p < 0.01), and STAT3 (*F*_*1,95*_ = 4.978, p < 0.05) expression, such that mice injected at ZT 14 displayed elevated responses to chemotherapy injection compared to animals injected at ZT 2. This interaction was not observed in hypothalamic IL-6. This expression profile is nearly the opposite pattern to that observed in the spleen (Fig. 3).

Chemotherapy did not affect IL-1β expression within the hippocampus (*F*_*1,90*_ = 0.548, p > 0.05). However, there was an interaction among treatment, injection time, and time after injection (*F*_*3,90*_ = 5.422, p < 0.005), such that mice injected at ZT 14 showing an increase in expression at 9 h after injection that was not evident in mice injected at ZT 2 (Fig. 4h). Similar results were obtained for hippocampal TNF-α, with no main effect of treatment evident (*F*_*1,90*_ = 0.063, p > 0.05), but an interaction between treatment and injection time (*F*_*1,90*_ = 10.954, p < 0.002), and an interaction among treatment, injection time, and hours after injection (*F*_*3,90*_ = 3.323, p < 0.05); i.e., animals injected at ZT 14 displayed increased hippocampal TNF-α 3 and 9 h post-injection (Fig. 4i).

### Time of Injection Alters the Transcription of Enzymes Involved in Cyclo/Dox Metabolism

**Figure 5:**
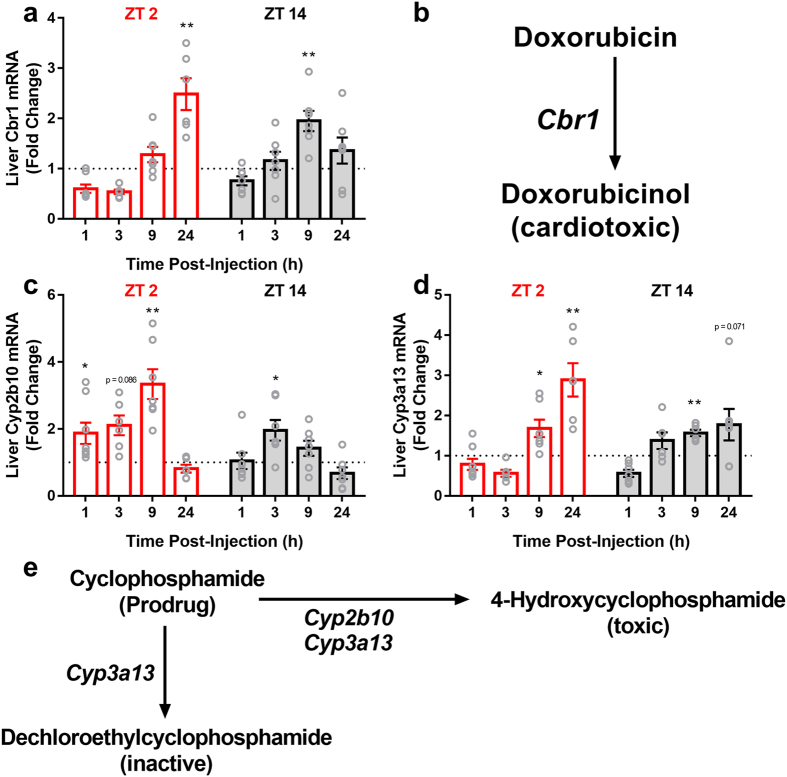
Time-of-day alters the transcriptional activity of enzymes involved in cyclo/dox metabolism. (**a**) Carbonyl reductase (Cbr1) showed differential expression following chemotherapy injection at ZT 2 or ZT 14. (**b**) this enzyme is responsible for the conversion of doxorubicin into a more toxic metabolite, doxorubicinol (DOX-OL). (**c**) Cyp2b10 was markedly elevated after ZT 2 injection, a pattern not evident after ZT 14 injection, (**d**) Cyp3a13 also showed a similar induction pattern dependent on time-of-day, (**e**) The prodrug cyclophosphamide is converted into a toxic metabolite (4-OH) via Cyp2b10 and cyp3a13. Alternatively, it is converted into an inactive product by cyp3a13 (DCE). (n = 5–8/group/timepoint; *p < 0.05, **p < 0.01 in relation to the time-matched vehicle control; Error bars represent S.E.M.) The dotted line at y = 1 represents normalized vehicle values.

**Figure 6:**
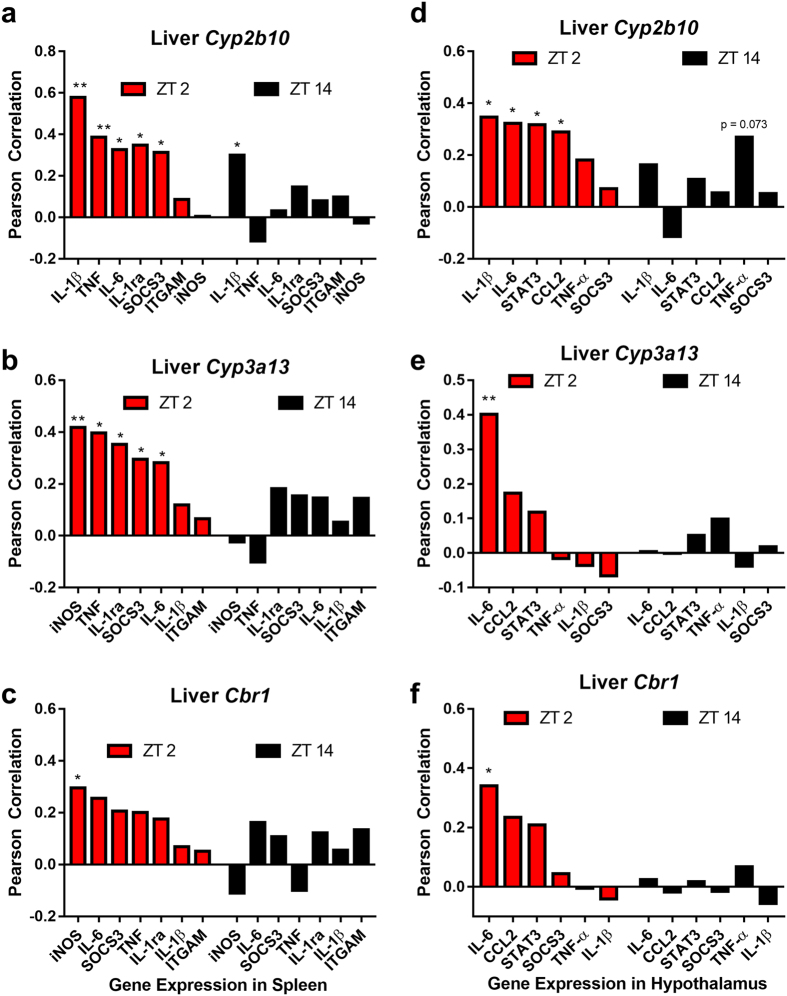
Splenic and hypothalamic inflammatory responses to chemotherapy are related to liver enzyme transcription only in mice injected at ZT 2. Pearson correlations between liver enzymes that metabolize cyclophosphamide: *Cyp2b10, Cyp3a13* and splenic (**a**,**b**) and hypothalamic (**d**,**e**) inflammatory mediators. *Cbr1*, responsible for doxorubicin metabolism and its relation to splenic (**c**) and hypothalamic (**f**) inflammatory gene transcription after chemotherapy at ZT 2 or ZT 14. (*p < 0.05, **p < 0.01).

The expression of Cytochrome p450 genes *Cyp2b10* and *Cyp3a13* as well as carbonyl reductase (*Cbr1*) were examined in the liver (Fig. 5). Chemotherapy significantly increased expression of Cyp2b10 (*F*_*1,93*_ = 25.666, p < 0.0001), Cyp3a13 (*F*_*1,90*_ = 12.035, p < 0.001), and Cbr1 (*F*_*1,95*_ = 7.131, p < 0.01) in the liver compared to vehicle injection. There also was an interaction between treatment and injection time in Cyp2b10 expression (*F*_*1,93*_ = 8.565, p < 0.005), an effect not evident in Cyp3a13 (p > 0.05) or Cbr1 (p > 0.05) expression. The activity of *Cyp2a10* positively correlated with the splenic expression of many proinflammatory cytokines in mice that were injected at ZT 2 (IL-6: R = 3.26, p < 0.05; IL-1β: R = 0.579, p < 0.0001; SOCS3: R = 0.314, p < 0.05; TNF-α: R = 0.387, p < 0.01; IL-1ra: R = 0.348, p < 0.05), but not in mice injected at ZT 14 (except IL-1β: R = 0.3, p < 0.05) (Fig. 6a–c). A similar pattern was observed for *Cyp3a13* (ZT 2: iNOS: R = 0.418, p < 0.01; SOCS3: R = 0.295, p < 0.05; TNF-α: R = 0.396, p < 0.01; IL-1ra: R = 0.353, p < 0.05; ZT 14: all correlations p > 0.05), but not for *Cbr1* (excepting ZT 2 iNOS: R = 0.295, p < 0.05; ZT 14: All genes p > 0.05). Liver transcription of these enzymes was related in a similar fashion to hypothalamic cytokine transcription, with *Cyp2b10* correlating with several genes in mice injected at ZT 2 (Fig. 6d–f) (IL-1β: R = 0.346, p < 0.05; IL-6: R = 0.323, p < 0.05; STAT3: R = 0.318, p < 0.05; CCL2: R = 0.29, p < 0.05), but not ZT 14 (p > 0.05 in all gene examined). *Cyp3a13* transcription was associated with hypothalamic IL-6 expression in mice injected at ZT 2 (R = 0.401, p < 0.01), but not ZT 14 (p > 0.05 for all genes examined). Likewise, liver *Cbr1* was positively correlated with hypothalamic IL-6 only in mice injected at ZT 2 (R = 0.34, p < 0.05; ZT 14: all genes examined p > 0.05).

### Time of Injection Does Not Alter Circulating Cytokine Levels

**Figure 7:**
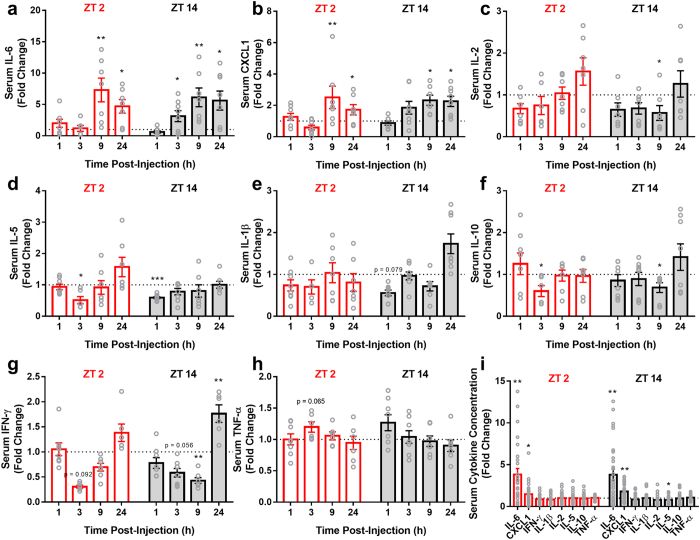
Serum (**a**) IL-6 and (**b**) CXCL1 concentrations were elevated in response to chemotherapy independent of time-of-day. (**c**) IL-2, (**d**) IL-5, (**e**) IL-1β, (**f**) IL-10, (**g**) IFN-γ, and (**h**) TNF-α were unchanged in response to treatment. (**i**) Average values between ZT 2 and ZT 14-injected animals over the entire post-injection time frame. (N = 4–8/group/timepoint; Error bars represent S.E.M., *p < 0.05, **p < 0.01 compared to the time-matched vehicle average) (the dotted line at y = 1 represents normalized vehicle values).

There was a significant increase in serum IL-6 (*F*_*1,94*_ = 38.485, p < 0.0001) and CXCL1 (aka KC/GRO) (*F*_*1,98*_ = 19.19, p < 0.0001) concentrations, but not IFN-γ, IL-10, IL-1β, IL-2, IL-5, or TNF-α (p > 0.05 in all cases), among chemo-treated mice compared to vehicle-treated mice. There was no interaction between injection time and treatment in any of the serum proteins examined (p > 0.05 in all cases) (Fig. 7).

## Discussion

Together, the data presented here demonstrate tissue-specific, time-of-day-dependent changes in inflammatory responses to cytotoxic chemotherapy. Mice injected with Cyclo/Dox at ZT 2 exhibited no increase in hypothalamic IL-1β, TNF-α, IL-6, CCL2, STAT3, or SOCS3, a downstream target of JAK/STAT signaling, compared to vehicle treated animals. Animals treated at ZT 14, however, showed a biphasic expression of these genes in the hypothalamus, with peaks at 3 and 24 h after injection (Fig. 4). This pattern was similar in the hippocampus, where animals injected at ZT 14 increased hippocampal IL-1β and TNF-α 3–9 h following injection, a pattern not evident in mice injected at ZT 2 (Fig. 4h,i). The induction of inflammatory mediators within the brain displayed the opposite pattern from the spleen (Fig. 4). This difference between peripheral and central inflammatory signaling in response to chemotherapy may depend on clock gene activity within brain microglia and peripheral macrophages, which contain autonomous clocks that are 6–8 h out of phase with each other^12^,^21^. Additionally, central clocks and those in peripheral organs are ~4 h out of phase, reflecting the master/slave oscillator relationship between them^22^,^23^. These data also highlight the notion that peripheral responses to an immune challenge cannot be considered a reliable indicator of the central response (e.g., see ref. 24). Results such as these provide additional evidence for the pleiotropic effects of cytokines, dependent on time-of-day and tissue specificity.

There also appears to be temporal variation in the activation of metabolic pathways of cyclophosphamide and doxorubicin. Cyclophosphamide is a prodrug that requires metabolic activation. *Cyp2b10* is responsible for the conversion of cyclophosphamide into a toxic metabolite, 4-hydroxycyclophosphamide (4-OH), whereas *Cyp3a13* contributes to both activation and inactivation of cyclophosphamide through conversion to 4-OH and the inactive compound dechloroethylcyclophosphamide (DCE; Fig. 5). The activity of these enzymes significantly contributes to the toxicity of cyclophosphamide, and influences its tolerability. In the present study, mice injected at ZT 2 had increased *Cyp2b10* gene expression (Fig. 5),which may suggest increased production of toxic 4-hydroxycyclophosphamide (4-OH) relative to mice treated at ZT 14. Consistent with this observation, a previous study reported increased mortality among mice treated with cyclophosphamide between ZT 22 and ZT 2 relative to mice treated between ZT 10 and ZT14^3^. This study, as well as a more recent one^16^, reported no temporal effects on cyclophosphamide metabolism, however, these studies did not examine enzyme activity or serum metabolite concentration later than 4 hours after injection, while in the present study, the temporal effect on the metabolic pathway did not emerge until >9 hours after injection (Fig. 5). We also observed a time-of-day effect in doxorubicin (DOX) metabolism. An initial step in DOX metabolism is its reduction to doxorubicinol (DOX-OL). DOX-OL is a less effective anti-neoplastic agent, and displays a greater capacity to induce cardiotoxicity. This initial conversion of DOX→DOX-OL is catalyzed by carbonyl reductase I (*Cbr1*), primarily in the liver^25^. Mice that received chemo at ZT 2 elevated *Cbr1* transcription at 24 h after injection compared to mice that received treatment at ZT 14 (Fig. 5a). Taken together, the data suggest that enhanced inflammation in mice injected at ZT 2 may reflect increased production of toxic drug metabolites 4-OH and DOX-OL. In support of this explanation, splenic inflammation was positively correlated with liver enzyme activity only in the mice administered Cyclo/Dox at ZT 2, but not ZT 14 (Fig. 6). This ZT 2 specific correlation was also observed within the hypothalamus (Fig. 6d–f), even though inflammation in this tissue was enhanced in mice injected at ZT 14 relative to ZT 2, indicating that liver metabolism of Cyclo/Dox may be uncoupled from inflammatory processes occurring in the brain. Together, these data support the hypothesis that time-of-day dependent effects of administration likely depend on downstream cyclophosphamide actions on the DNA damage response, inflammation, or additional pathways^16^.

A potential contributor to the phenotype we observed could be sleep disruption. We have previously reported marked sleep fragmentation after administration of the same chemo regimen used in the present study^10^. As sleep disruption elicits an inflammatory response^26^,^27^, this may have contributed to the enhanced splenic inflammation observed in animals injected at ZT 2. Additionally, glucocorticoid concentrations peak during the early active phase in nocturnal rodents^28^, and they are negative regulators of inflammation, acting through multiple mechanisms^29^,^30^. Therefore, elevated baseline concentrations of glucocorticoids in mice injected at ZT 14 may have contributed to the dampened inflammatory response observed in the spleen, a pattern observed in the present study (Fig. 3i). Indeed, morning administration of doxorubicin and evening administration of cisplatin is much better tolerated than the opposite administration schedule in humans with advanced ovarian cancer^31^; it is hypothesized that peak glucocorticoid concentrations at the time of doxorubicin administration may buffer the inflammatory response to the drug.

Periphery-to-brain inflammatory signaling can be accomplished through several mechanisms^32^. Because we did not observe a change in serum cytokine responses to chemotherapy dependent on time-of-day (Fig. 7), it is unlikely that humoral signaling is the primary means by which chemotherapy elicits differential hypothalamic effects at ZT 2 and ZT 14. Alternatively, transcription of the monocyte recruiting chemokine CCL2 in the hypothalamus was elevated following administration of chemotherapy at ZT 14, but not ZT 2 (Fig. 4f). Recently, direct interactions among the circadian clock and CCL2 have been reported to mediate time-of-day dependent responses to peripheral endotoxin administration, with peak hypothalamic CCL2 expression during the dark (inactive) phase^33^. Therefore, the phenotype we observed within the hypothalamus may be driven by time-of-day dependent recruitment of peripheral monocytes/macrophages into the brain.

Several intriguing questions remain regarding the interactions among chemotherapy and other cancer- and treatment-related factors. For example, we chose to begin this line of inquiry by examining the effects of chemotherapy in non-tumor bearing mice because cyclophosphamide and doxorubicin combination therapy is typically given in an adjuvant setting once the tumor has been resected^19^. However, additional studies are necessary to determine whether *a prior* or current tumor sensitizes or causes a temporal shift in inflammatory responses to doxorubicin and cyclophosphamide. Comparing responses in mice that are tumor-naïve, tumor-bearing, versus tumor-resected will be critical for assessing whether tumors and/or the surgery associated with their removal prime inflammatory responses to chemotherapy and if so, how long after tumor removal the effects persist. Indeed, the primary reason the current study was conducted in BALB/C mice is that there are well-characterized metastatic and non-metastatic mammary tumor cell lines that are specific to this mouse strain (e.g., 4T1, 67NR, 4T07). Despite muted nightly melatonin rhythms^34^, BALB/C mice display robust rhythms in immune cell activity, cytokine secretion, and response to endotoxin challenge. For instance, BALB/C mice display robust time-of-day dependent changes in LPS-responsiveness dependent on diurnal variations in NF-κB activity^35^. Additionally, circadian sensitivity to cyclophosphamide has been demonstrated in C57BL/6j mice, which also lack robust circadian rhythms in melatonin production and secretion^3^,^34^, suggesting that a robust melatonin signal is not required for the time-of-day dependent effects of cyclophosphamide. Regardless, further studies should investigate a potential role for endogenous melatonin in buffering inflammatory responses to cytotoxic chemotherapy.

The predictive value of rodent studies for human inflammatory conditions also has become a topic of debate recently^36^,^37^,^38^. How closely the liver, spleen, and brain inflammatory gene expression data collected in mice in the current study reflect changes occurring in human cancer patients is not ethically determinable with currently available technology. However, our data demonstrate that the *potential* exists for circadian differences in inflammatory and metabolic responses to a common chemotherapy regimen, and also that it should not be assumed that all tissues respond similarly. Nonetheless, the temporal aspects of chemotherapy treatment in patients with breast cancer is worth exploring given that there is a growing literature demonstrating diurnal variation in immune parameters associated with inflammatory diseases and experimental endotoxin exposure in people^39^,^40^.

In sum, our data demonstrate time-of-day-dependent changes in inflammatory responses to cytotoxic chemotherapy. Specifically, splenic inflammatory responses to cyclophosphamide and doxorubicin are maximal when the drugs are administered during the early inactive phase (ZT 2), and lowest when administered during the early active phase (ZT 14). By contrast, inflammatory gene expression within the hypothalamus displays the reciprocal pattern, as mice injected at ZT 14 show an enhanced hypothalamic inflammatory response compared to those injected at ZT 2. Additionally, the activity of enzymes responsible for the conversion of doxorubicin and cyclophosphamide into their toxic metabolites displays differential induction dependent on time-of-day, and their activity correlates with splenic and hypothalamic inflammatory responses only in mice injected during the early inactive phase. These data further add to the growing literature on cancer ‘chronotherapy’, and highlight inflammatory responses to treatment as a proximate cause of chemotherapy-induced side effects dependent on time of drug administration.

## Additional Information

**How to cite this article**: Borniger, J. C. *et al*. Time-of-Day Dictates Transcriptional Inflammatory Responses to Cytotoxic Chemotherapy. *Sci. Rep.* **7**, 41220; doi: 10.1038/srep41220 (2017).

**Publisher's note:** Springer Nature remains neutral with regard to jurisdictional claims in published maps and institutional affiliations.

## Figures and Tables

**Figure 1:**
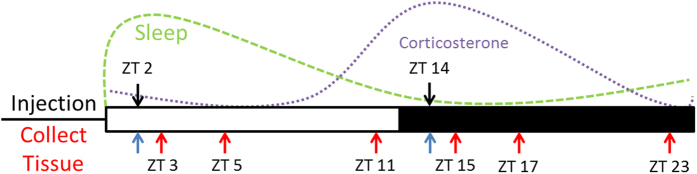
Experimental Design. Mice were intravenously injected with Cyclo/Dox or the vehicle at ZT 2 or ZT 14. Tissue was collected 1, 3, 9, and 24 h later from each of these time points. Blue arrows denote the 24 h tissue collection time point.

**Table 1:** Primer/probe information for targets used in qPCR.

Gene	Assay ID	Amplicon Size (bp)
*Il6*	Mm00446190_m1	78
*Il1b*	Mm00434228_m1	90
*Socs3*	Mm01249143_g1	113
*Tnf*	Mm00443258_m1	81
*Il1rn*	Mm00446186_m1	80
*Cbr1*	Mm04207333_g1	83
*Cyp2b10*	Mm00456588_mH	88
*Cyp3a13*	Mm00484110_m1	88
*Itgam*	Mm00434455_m1	69
*Stat3*	Mm01219775_m1	75
*Nos2 (iNos)*	Mm00440502_m1	66
*Ccl2*	Mm00441242_m1	74
*18s*	Hs99999901_s1	187

## References

[b1] Lévi F., Okyar A., Dulong S., Innominato P. F. & Clairambault J. Circadian timing in cancer treatments. Annu Rev Pharmacol Toxicol 50, 377–421 (2010). 20055686 10.1146/annurev.pharmtox.48.113006.094626

[b2] Granda T. G. . Experimental chronotherapy of mouse mammary adenocarcinoma MA13/C with docetaxel and doxorubicin as single agents and in combination. Cancer Res 61, 1996–2001 (2001). 11280758

[b3] Gorbacheva V. Y. . Circadian sensitivity to the chemotherapeutic agent cyclophosphamide depends on the functional status of the CLOCK/BMAL1 transactivation complex. Proc Natl Acad Sci USA 102, 3407–12. (2005). 15689397 10.1073/pnas.0409897102 PMC546637

[b4] Sothern R., Halberg F. & Hrushesky W. Circadian stage not time-of-day characterizes doxorubicin susceptibility rhythm of mice in continuous light. Annu Rev Chronopharmacol 5, 385 (1988).

[b5] Levi F. & Schibler U. Circadian rhythms: mechanisms and therapeutic implications. Annu Rev Pharmacol Toxicol 47, 593–628 (2007). 17209800 10.1146/annurev.pharmtox.47.120505.105208

[b6] Weymann K. B., Wood L. J., Zhu X. & Marks D. L. A role for orexin in cytotoxic chemotherapy-induced fatigue. Brain Behav Immun 37, 84–94 (2014). 24216337 10.1016/j.bbi.2013.11.003 PMC3951615

[b7] Smith L. B. . The role of IL-1β and TNF-α signaling in the genesis of cancer treatment related symptoms (CTRS): a study using cytokine receptor-deficient mice. Brain Behav Immun 38, 66–76 (2014). 24412646 10.1016/j.bbi.2013.12.022 PMC3989411

[b8] Elsea C. R., Roberts D. A., Druker B. J. & Wood L. J. Inhibition of p38 MAPK Suppresses Inflammatory Cytokine Induction by Etoposide, 5-Fluorouracil, and Doxorubicin without Affecting Tumoricidal Activity. PLoS ONE 3, e2355, doi: 10.1371/journal.pone.0002355 (2008). 18523641 PMC2396285

[b9] Wong J. . Small molecule kinase inhibitors block the ZAK-dependent inflammatory effects of doxorubicin. Cancer Biol Ther 14, 56–63 (2013). 23114643 10.4161/cbt.22628 PMC3566053

[b10] Borniger J. C., Gaudier-Diaz M. M., Zhang N., Nelson R. J. & DeVries A. C. Cytotoxic chemotherapy increases sleep and sleep fragmentation in non-tumor-bearing mice. Brain Behav Immun 47, 218–27 (2015). 25449581 10.1016/j.bbi.2014.11.001

[b11] Guan Z. . Interleukin-6 levels fluctuate with the light–dark cycle in the brain and peripheral tissues in rats. Brain Behav Immun 19, 526–9 (2005). 16214023 10.1016/j.bbi.2005.01.005

[b12] Fonken L. K. . Microglia inflammatory responses are controlled by an intrinsic circadian clock. Brain Behav Immun 45, 171–9 (2015). 25433170 10.1016/j.bbi.2014.11.009 PMC4386638

[b13] Coogan A. N. & Wyse C. A. Neuroimmunology of the circadian clock. Brain Res 1232, 104–12 (2008). 18703032 10.1016/j.brainres.2008.07.087

[b14] Besedovsky L., Lange T. & Born J. Sleep and immune function. Eur J Physiol 463, 121–37 (2012). 10.1007/s00424-011-1044-0 PMC3256323 22071480

[b15] Scheving L. E., Burns E. R., Pauly J. E. & Halberg F. Circadian bioperiodic response of mice bearing advanced L1210 leukemia to combination therapy with adriamycin and cyclophosphamide. Cancer Res 40, 1511–1515 (1980). 7370988

[b16] Van Dycke K. C. . A day and night difference in the response of the hepatic transcriptome to cyclophosphamide treatment. Arch Toxicol 89, 221–31 (2015). 24819615 10.1007/s00204-014-1257-z

[b17] Poniatowski B. C., Grimm P. & Cohen G. Chemotherapy-induced menopause: a literature review. Cancer Invest 19, 641–8 (2001). 11486707 10.1081/cnv-100104292

[b18] Molina J. R., Barton D. L. & Loprinzi C. L. Chemotherapy-induced ovarian failure. Drug Safety 28, 401–16 (2005). 15853442 10.2165/00002018-200528050-00004

[b19] Shulman L. N. . Comparison of doxorubicin and cyclophosphamide versus single-agent paclitaxel as adjuvant therapy for breast cancer in women with 0 to 3 positive axillary nodes: CALGB 40101 (Alliance). J Clin Oncol 32, 2311–2317 (2014). 24934787 10.1200/JCO.2013.53.7142 PMC4105484

[b20] Reagan-Shaw S., Nihal M. & Ahmad N. Dose translation from animal to human studies revisited. FASEB J 22, 659–61 (2008). 17942826 10.1096/fj.07-9574LSF

[b21] Keller M. . A circadian clock in macrophages controls inflammatory immune responses. Proc Natl Acad Sci USA 106, 21407–12 (2009). 19955445 10.1073/pnas.0906361106 PMC2795539

[b22] Balsalobre A., Damiola F. & Schibler U. A serum shock induces circadian gene expression in mammalian tissue culture cells. Cell 1998 93, 929–37 (1998). 9635423 10.1016/s0092-8674(00)81199-x

[b23] Balsalobre A. Clock genes in mammalian peripheral tissues. Cell Tissue Res 309, 193–9 (2002). 12111549 10.1007/s00441-002-0585-0

[b24] Karelina K. . Social isolation alters neuroinflammatory response to stroke. Proc Natl Acad Sci USA 106, 5895–900 (2009). 19307557 10.1073/pnas.0810737106 PMC2667090

[b25] Kassner N. . Carbonyl reductase 1 is a predominant doxorubicin reductase in the human liver. Drug Metab Dispos 362, 113–20 (2008). 10.1124/dmd.108.022251 18635746

[b26] Faraut B., Boudjeltia K. Z., Vanhamme L. & Kerkhofs M. Immune, inflammatory and cardiovascular consequences of sleep restriction and recovery. Sleep Med Rev 16, 137–49 (2012). 21835655 10.1016/j.smrv.2011.05.001

[b27] Ashley N. T., Sams D. W., Brown A. C. & Dumaine J. E. Novel environment influences the effect of paradoxical sleep deprivation upon brain and peripheral cytokine gene expression. Neurosci Lett 615, 55–59 (2016). 26806035 10.1016/j.neulet.2016.01.013 PMC4755797

[b28] Sundar I. K. . Serotonin and corticosterone rhythms in mice exposed to cigarette smoke and in patients with COPD: implication for COPD-associated neuropathogenesis. PLOS ONE 9,: e87999 (2014). 24520342 10.1371/journal.pone.0087999 PMC3919731

[b29] Scheiermann C., Kunisaki Y. & Frenette P. S. Circadian control of the immune system. Nat Rev Immunol 13, 190–8 (2013). 23391992 10.1038/nri3386 PMC4090048

[b30] Barnes P. J. Anti-inflammatory actions of glucocorticoids: molecular mechanisms. Clin Sci 94, 557–72 (1998). 9854452 10.1042/cs0940557

[b31] Hrushesky W. J. Circadian timing of cancer chemotherapy. Science 228, 73–75 (1985). 3883493 10.1126/science.3883493

[b32] Quan N. & Banks W. A. Brain-immune communication pathways. Brain Behav Immun 21, 727–35 (2007). 17604598 10.1016/j.bbi.2007.05.005

[b33] Duhart J. M., Brocardo L., Fedele M. L., Guglielmotti A. & Golombek D. A. CCL2 mediates the circadian response to low dose endotoxin. Neuropharmacology 108, 373–81 (2016). 27178133 10.1016/j.neuropharm.2016.05.005

[b34] Vivien-Roels B. . Daily variations in pineal melatonin concentrations in inbred and outbred mice. J Biol Rhythms 13, 403–409 (1998). 9783231 10.1177/074873098129000228

[b35] Spengler M. L. . Core circadian protein CLOCK is a positive regulator of NF-κB–mediated transcription. Proc Natl Acad Sci USA 109, E2457–2465 (2012). 22895791 10.1073/pnas.1206274109 PMC3443185

[b36] Seok J. . Genomic responses in mouse models poorly mimic human inflammatory diseases. Proc Natl Acad Sci USA 110, 3507–3512 (2013). 23401516 10.1073/pnas.1222878110 PMC3587220

[b37] Takao K. & Miyakawa T. Genomic responses in mouse models greatly mimic human inflammatory diseases. Proc Natl Acad Sci USA 112, 1167–1172 (2015). 25092317 10.1073/pnas.1401965111 PMC4313832

[b38] Shay T., Lederer J. A. & Benoist C. Genomic responses to inflammation in mouse models mimic humans: we concur, apples to oranges comparisons won’t do. Proc Natl Acad Sci USA 112, E346 (2015). 25540423 10.1073/pnas.1416629111 PMC4313855

[b39] Alamili M., Bendtzen K., Lykkesfeldt J., Rosenberg J. & Gögenur I. Pronounced inflammatory response to endotoxaemia during nighttime: a randomised cross-over trial. PloS One 9, e87413 (2014). 24475284 10.1371/journal.pone.0087413 PMC3903723

[b40] Petrovsky N., McNair P. & Harrison L. C. Diurnal rhythms of pro-inflammatory cytokines: regulation by plasma cortisol and therapeutic implications. Cytokine 10, 307–312 (1998). 9617577 10.1006/cyto.1997.0289

